# MLDAAPP: machine learning data acquisition for assessing population phenotypes

**DOI:** 10.1093/g3journal/jkaf173

**Published:** 2025-08-01

**Authors:** Amir R Gabidulin, Seth M Rudman

**Affiliations:** School of Biological Sciences, Washington State University, 14204 NE Salmon Creek Avenue, Vancouver, WA 98686, United States; School of Biological Sciences, Washington State University, 14204 NE Salmon Creek Avenue, Vancouver, WA 98686, United States

**Keywords:** computer vision, machine learning, *Drosophila*, phenotype, YOLOv8, phenomics

## Abstract

Collecting phenotypic data from many individuals is critical to answering fundamental biological questions, particularly in genetics. Yet, whole organismal phenotypic data are still often collected manually; limiting the scale of data generation, precluding reproducible workflows, and creating potential bias. Computer vision can ameliorate these issues, but currently available packages only operate with specific inputs and have limited scalability. We present Machine Learning Data Acquisition for Assessing Population Phenotypes (MLDAAPP), a package of tools built on YOLOv8 for collecting phenotypic data from groups of individuals that is flexible for generating counts (e.g. *Drosophila* fecundity, census), movement (e.g. activity, aversive behavior), and many other potential phenotypes. MLDAAPP is both accurate and uniquely effective for obtaining phenotypic data in challenging conditions—particularly images and videos of varying quality derived from both lab and field environments. Employing MLDAAPP solves key issues of reproducibility and increases the scale and scope of data generation.

## Introduction

Biological measurements on groups of individuals, including counts, sizes, and patterns of movement, are essential data across a range of disciplines including ecology, evolutionary biology, animal behavior, and genetics (McGill et al. 2006; Bilder et al. 2009; Houle et al. 2010; Arnegard et al. 2014). Collection of these data is still primarily done manually, which limits both the scope and reproducibility of the resultant outputs (Schneider et al. 2012; Weinstein 2018; Lürig et al. 2021). The rapid growth of machine learning (ML) is potentially transformative to the way data is collected and ML is successfully employed in cell biology and several areas of health science research for data generation (Ching et al. 2018; Moen et al. 2019). The automated extraction of information from images and videos, often called computer vision (CV), is a particularly promising area for further streamlining biological data generation (Lürig et al. 2021; Orenstein et al. 2022). Existing ML programs present considerable barriers to the collection of whole-organism phenotypic data. These include monetary cost, difficult, or time-consuming pathways to implementation, and designs optimized for the collection of data only under very specific circumstances (Lürig et al. 2021; Schneider et al. 2023). These challenges can be insurmountable when researchers seek to use ML methods to collect phenotypic data in nonstandard assays, field research environments, simultaneously for many individuals, or using existing photo and video inputs (Weinstein 2018; Romero-Ferrero et al. 2019; Schneider et al. 2023).

CV methods are currently used to generate datasets in ecology, evolution, and genetics (Romero-Ferrero et al. 2019; Lürig et al. 2021; Walter and Couzin 2021; Lürig 2022). These methods employ background subtraction to allow for a streamlined approach to analyzing photo and video data. The measurement of fecundity in *Drosophila*, which is both frequently done and time-consuming, has been the focus of CV applications (Ng’oma et al. 2018; Nouhaud et al. 2018). Yet, CV can be done with supervised ML approaches in which a model is trained with a user-annotated training dataset and subsequently employed to make weighted decisions to create bounding boxes or instance segmentation masks around objects. Supervised ML approaches allow users to define what to detect rather than using an *a priori* dictated set of rules as is found in pure background subtraction approaches. This increased flexibility is tremendously beneficial when phenotypic data are collected outside laboratory environments, across a range of assay conditions, or using input images or videos without optimized contrast (Weinstein 2018).

**Fig. 1.**
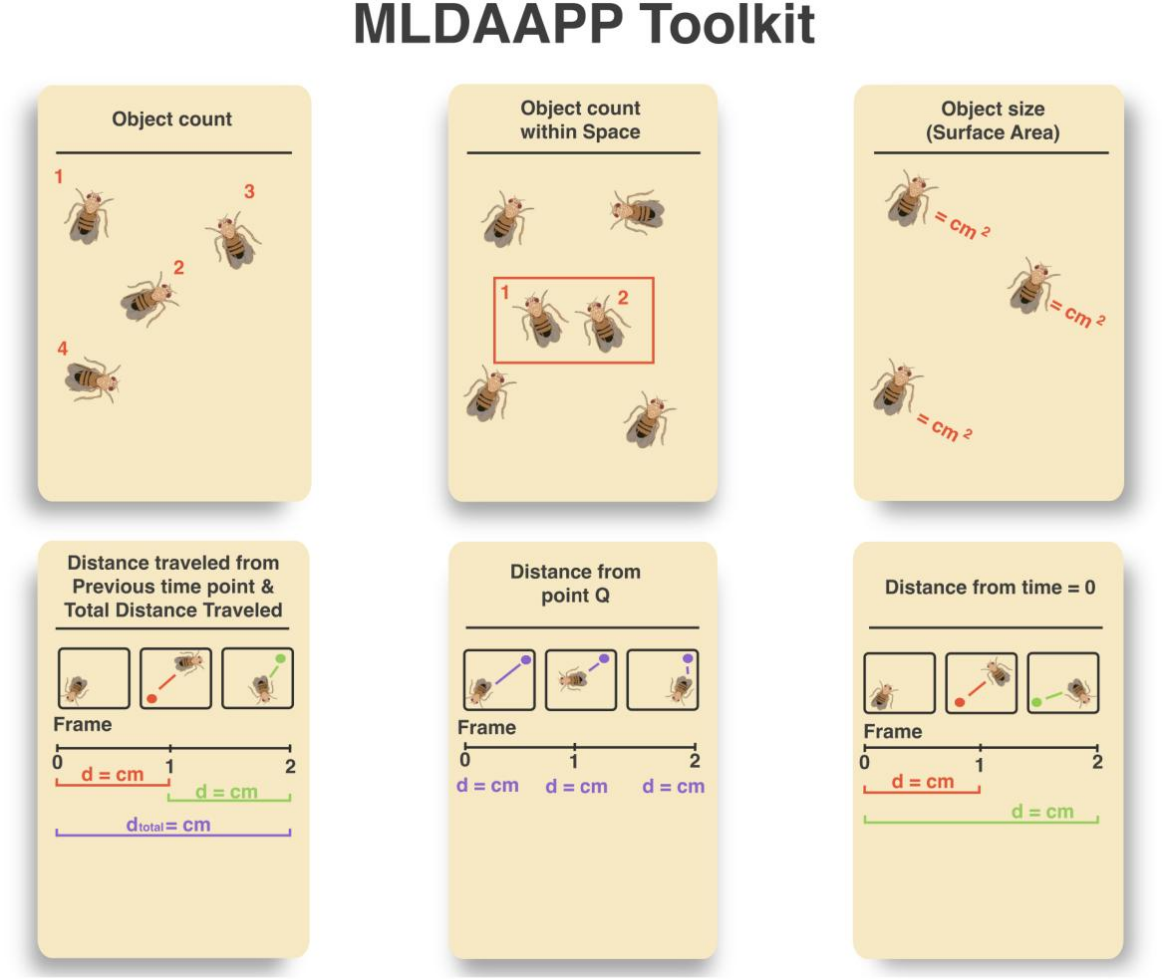
Visual demonstrations of key outputs that can be generated using user-trained computer vision models in MLDAAPP. These include: object counts, object counts within user-defined bounding boxes, object size (surface area), distance traveled (over particular time intervals and total), distance from a user-defined reference point, and distance from the starting point. Additional utilities can be found on the GitHub page.

**Fig. 2.**
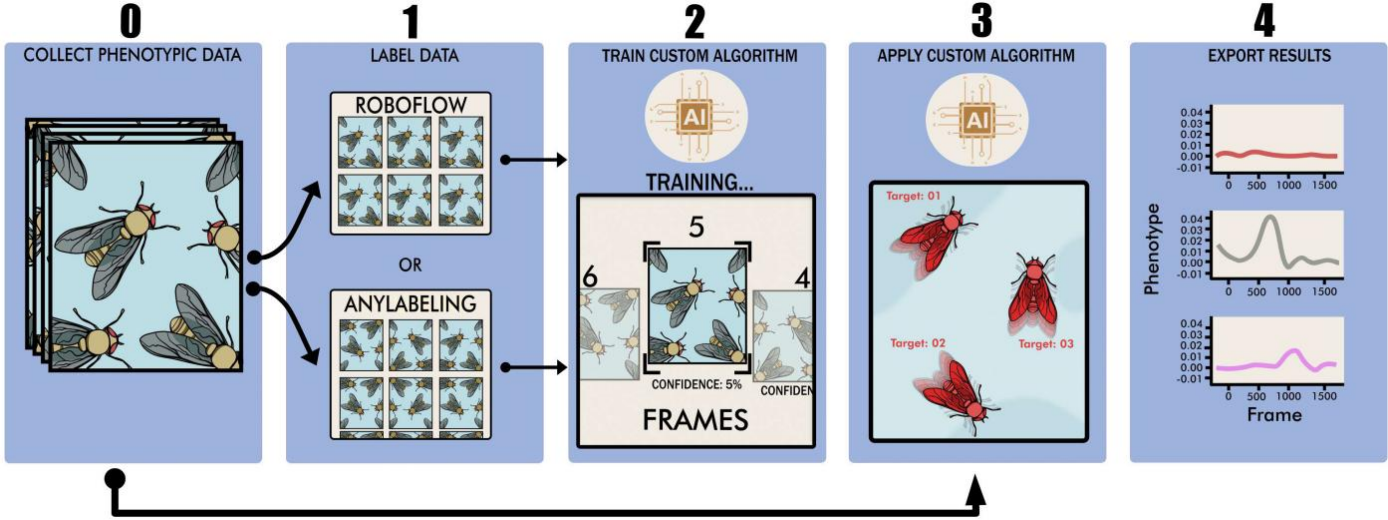
A pictorial guide to the workflow of implementing MLDAAPP for the collection of phenotypic data from groups of individuals. Step 0—create images or videos of the desired phenotype to be quantified. As a general rule, having individuals large enough in the frame to be readily identified by the human eye is necessary for accuracy. Step 1—annotate the inputs to create a training dataset for YOLOV8 using either a feature-rich annotation web-service (RoboFlow) or a locally run labeling software (AnyLabeling). Step 2 using the annotated dataset to train and verify the accuracy of a custom YOLOV8 model. Step 3—apply the custom YOLOV8 algorithm to the desired dataset. Step 4—use MLDAAPP tools to translate YOLOV8 outputs into phenotypic data for analysis.

Here, we introduce MLDAAPP, a package of scripts, implementation guidelines, and annotated training sets that leverages existing ML tools for the collection of phenotypic data. The purpose of this package is to streamline the process of the collection of common phenotypic data via CV for a range of taxa, but with specific tool development for implementation in the study of *Drosophila*. MLDAAPP is focused on user simplicity, flexibility, and precision and utilizes an open-source ML product, YOLOv8, based on enterprise level supervised ML and computer vision algorithms that offers state-of-the-art performance in speed and accuracy (Jocher et al. 2023). MLDAAPP assists in translating YOLOv8 to quantify photos and videos to produce object counts, object sizes, positions, avoidance/preference behaviors, and locomotor activity with a focus on data outputs that are simple to analyze for biologists (Fig. 1). Implementing MLDAAPP for data collection requires little to no upfront cost and provides considerably more flexibility than other CV programs (Fig. 2).

## Methods

A comprehensive user guide, detailing image and video input generation, annotation of user-defined training sets, model training, pretrained models with associated input collection protocols, and implementation of YOLOv8 is available on the MLDAAPP GitHub (https://github.com/ganamir/MLDAAPP).

### Input preparation, model training, model implementation, and MLDAAPP utility

Crucial to the implementation of MLDAAPP is the preparation of the inputs for the CV algorithm, either photos or videos, which are used to train the custom detection model. Lürig et al. (2021) provide both a history of CV for the collection of phenotypic data for biology and general best practices for input preparation that largely apply to the use of MLDAAPP.

**Fig. 3.**
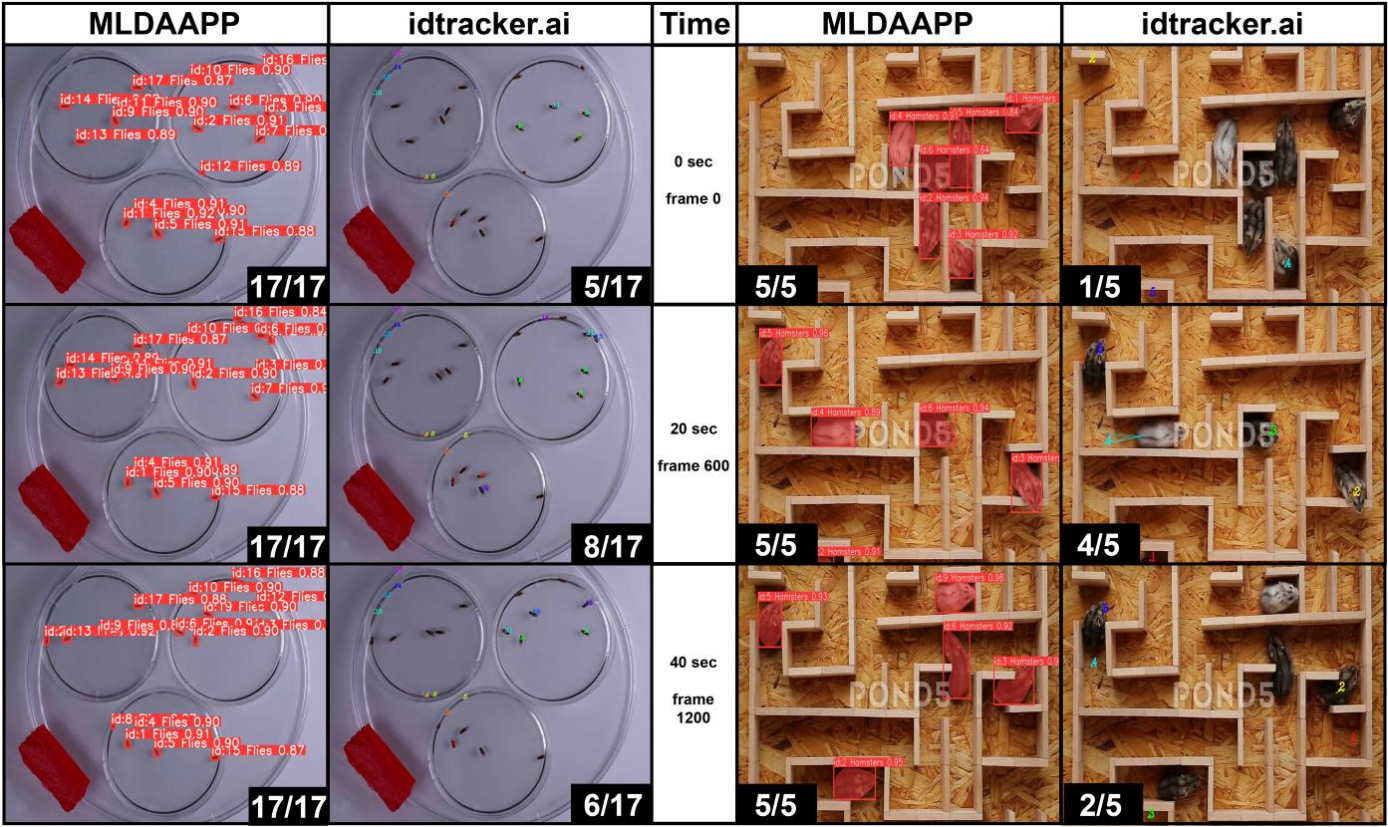
Model comparison between MLDAAPP and idtracker.ai using nonstandardized environments with the number of individuals tracked/all of the individuals in the frame at the bottom of each frame (Supplementary Table 1). The left side shows sequential video frames tracking *Drosophila* activity with all individuals tracked in MLDAAPP and consistently >50% of individuals tracked in idtracker.ai. The right side shows results of tracking hamsters in a maze with MLDAAPP, again showing superior tracking. These videos were also conducted using TRex (Walter and Couzin 2021), which performed similarly to idtracker.ai.

Generally, if a human is able to detect the object within the frame of the input without magnification, then it is likely possible to train the algorithm to do the same (Fig. 3). Once inputs are collected, models need to be trained by user annotation. The MLDAAPP GitHub page describes 2 methods to create an annotated training set, a locally run labeling software (for those with sensitive data—AnyLabeling), and a feature-rich web-service (Roboflow). Briefly, model accuracy is strongly impacted by annotation and it is important to include a representative sample across the full range of inputs in training sets. Model accuracy in object detection is typically evaluated by precision (true positives/true positives + false positives), recall (true positives/true positives + false negatives), and mean average precision which is composite of precision and recall across a range of conditions. These values are calculated by a comparison of a “validation” set that is a subset of the full trained annotation set. Anecdotally, training sizes of ∼100 annotated inputs are reasonably accurate, though this is highly dependent. As a general guide, we have found that across a range of trained datasets spanning organisms and environmental conditions, precision, recall, and mean average precision of 70–100% are accurately enough to mimic human counts. For more detailed descriptions of model performances, we provide a table of model accuracy metrics for each pretrained model in the GitHub.

Once annotations are complete, they are used to train a YOLOv8 model. To ensure an accurate model, it is crucial to assess model fit by experimenting with validation vs test splits of your data. Across our test cases, models built with 70/30 and 80/20 validation/test ratios of training inputs both yielded near identical mean average precision metrics when working within challenging sample datasets (Supplementary Fig. 1). The choice of YOLOv8 model size does impact the speed and accuracy of the detection; we recommend the usage of YOLOv8x (extra-large-model) for best results. Supplementary Table 2 briefly summarizes the most successful strategies in working with YOLOv8 and Botsort Tracker, an ML-based tracking algorithm that was used as an assistant in tracking objects throughout the varying trials.

Once the YOLVv8 model is successfully trained, MLDAAPP can be used to translate outputs from YOLOv8 for empirical research for object detection/tracking, instance segmentation, or be used in custom scripts for the extraction of relevant biological data (Fig. 1; Supplementary Figs. 3–6). MLDAAPP provides guidance on the generation of inputs and training sets, streamlining training of YOLOv8 algorithms, and transformations of YOLOv8 outputs into usable phenotypic information (Fig. 2). In addition, we provide pretrained algorithms for measured *Drosophila* fecundity and movement on the associated GitHub.

### Flexible CV method for the collection of *Drosophila* fecundity data and field counts

**Fig. 4.**
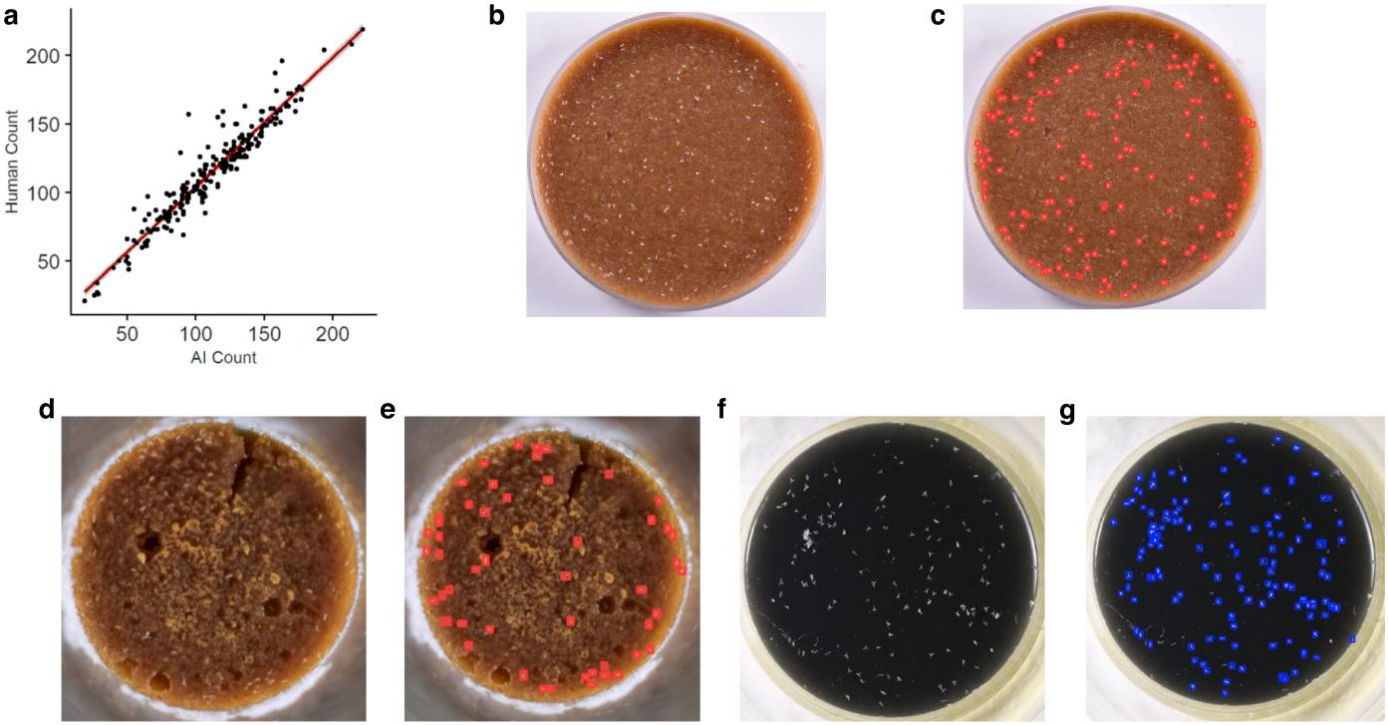
a) *Drosophila melanogaster* egg counts as measured by MLDAAPP vs Human counts in fecundity assay described in the methods. Line shows linear regression of this relationship. b) and c) A representative image from a *D. melanogaster* fecundity trial before and after annotation with MLDAAPP (respectively) on brown fecundity collection method (recipe in GitHub) is shown. d) and e) A similar protocol as b) and c) is shown, using narrow *Drosophila* vials. f) and g) The charcoal fecundity collection method (recipe in GiHub) is shown.

We tested the utility of YOLOv8 and MLDAAPP for generating phenotypic data from pictures and videos across conditions and taxa. First, we focused on generating fecundity data for *Drosophila*, which are often counted manually (Durham et al. 2014), but has also been the focus of several previous CV programs with very specific input generation procedures (Waithe et al. 2015; Ng’oma et al. 2018; Gomez et al. 2024). We tested MLDAAPP for collecting *Drosophila* fecundity using 2 photo input generation procedures, conducted by different research labs (Fig. 4). Details for photo input collection are provided in the GitHub. Briefly, we generated a dataset of 210 unique images of 35 mm petri dishes in which 5 females had been allowed to lay eggs for a 24 h period. We manually annotated images in a web-annotating service (details in GitHub) to create an ML model and we employed a “mosaic” digital augmentation, bolstering our data set to 504 images, and training on an 88–13% split for 100 epochs (see Supplementary Fig. 2 for details on the efficacy of this approach). We compare results to manual counts in both speed and accuracy.

**Fig. 5.**
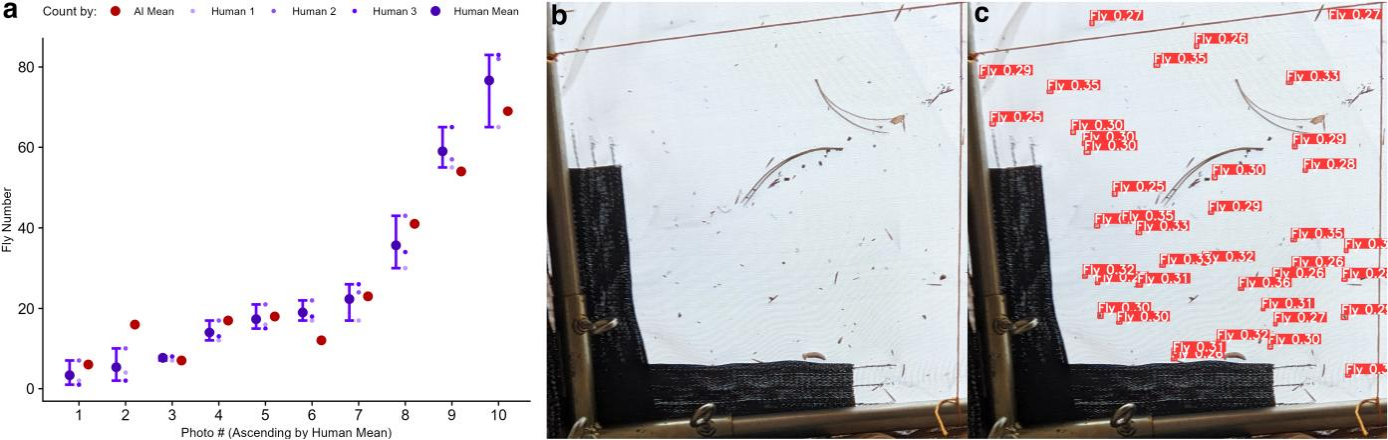
a) *Drosophila melanogaster* adult counts from outdoor population census photos as determined by MLDAAPP compared with manual counts produced by 3 different people. MLDAAP counts are in red and mean human counts are in blue (∓ SE) with counts from each individual represented by a dotted line. b) and c) A representative image from an area of an outdoor experimental cage with *D. melanogaster* before and after annotation with MLDAAPP (respectively) is shown.

Applications of CV have been challenging outside of highly controlled conditions, as background subtraction methods are limited in their accuracy with image noise. To test the utility of MLDAAPP for generating data from field contexts, we generated census photos from *Drosophila* field experiments (Rudman et al. 2019, 2022; Grainger et al. 2021). We used MLDAAPP to count *Drosophila* in field conditions by training a custom “census” detection model of 80 images of a known surface area from outdoor experimental arenas as seen in Fig. 5. Census photos were taken by separate individuals with their personal phones, requiring no investment into specialized equipment and providing maximum amount of flexibility. To validate the results, MLDAAPP outputs were compared with manually counted images. The model was trained with a data set of 80 photos using 70–30% split for 200 epochs (the passing of training data through the algorithm), where no physical or digital augmentations were used to enhance the training set.

### Movement tracking and comparison with existing CV models across taxa

MLDAAPP has also been optimized to track and extract movement data in video files across a range of backgrounds, group sizes, and movement assays in a wide range of taxa. The procedure to generate input video files is flexible to taxa and use case. We provide both details for data generation of a pretrained CV algorithm on the associated GitHub. Model implementation is challenging in videos that have low resolution, low framerate, and small object size (see Supplementary Table 1 “*Daphnia*”; Shahmohamadloo et al. 2024). These conditions initially rendered object tracking unreliable as individual IDs were frequently re-assigned. This problem was largely solved by increasing the amount of frames per second which allowed for a smoother object tracking with fewer object losses. Users should consider object size within the field of view, movement rate of the organism, and camera frame rate when creating inputs as each is impactful in the ability to extract phenotypic data using CV.

Overall, we found MLDAAPP was effective using a wide range of input videos to generate data on individual movement. There are existing packages for generating movement data from groups of individuals. We compared the efficacy of MLDAAPP with 3 such software packages, idTracker.ai, TRex and DeepLabCut, with several types of input videos ranging across taxa and video clarity. These included optimized videos provided by idTracker.ai, lower quality and lower material cost video inputs we collected, and challenging videos (“hamsters” and “honeybees”) taken from other sources.

## Results

### Data acquisition and interpretation of examples

To test MLDAAPP, we generated novel photo and video inputs across a range of conditions using *Drosophila* and sought out videos and photos from a range of taxa and testing conditions. We then annotated training sets and confirmed that the implementation of MLDAAPP scripts and supervised CV works across a range of taxa. Below, we provide results of case studies on implementing 3 ML data acquisition procedures, including an explicit comparison of object tracking with existing software packages for tracking—TRex (Walter and Couzin 2021), idtracker.ai (Romero-Ferrero et al. 2019), and DeepLabCut (Mathis et al. 2018; Moen et al. 2019; Lürig et al. 2021).

### Object counting case #1: Drosophila fecundity

We tested the implementation of YOLOv8 using MLDAAPP in automating the collection of fecundity data for *Drosophila melanogaster* (Fig. 4). In comparison to human counts done using a microscope model, accuracy overall was high (*R*^2^ = 0.91), even with inputs obtained using a simple imaging setup that produces some shadows and reflection (Fig. 4). Time savings were also substantial, particularly in cases of high egg density or large number of input measures. Training this high accuracy algorithm took ∼10 h, much of which was the time to generate image inputs. Though dependent on many factors, time savings likely start at ∼250 inputs for images and becomes considerable as inputs increase.

### Object counting case #2: *Drosophila* field photos

We determined that ML methods that employ custom annotations are also flexible enough to be used in natural environments. Using photographs as input data, we employed YOLOv8 and MLDAAPP toolset for census estimates of *Drosophila* from field experiments (Fig. 5). Figure 5a indicates the accuracy of the AI model, given 10 census pictures in comparison to 3 independent human counts. AI model counts were positively correlated with human 1 (*R*^2^ = 0.97, *P* < 0.001), human 2 (*R*^2^ = 0.98, *P* < 0.001), and human 3 (*R*^2^ = 0.97, *P* < 0.001) despite considerable image complexity (Fig. 5b). Due to considerable background complexity, larger frame of interest, image noise, and debris looking similar to a *Drosophila melanogaster*, these images are marginally more difficult to assess as a human, requiring magnification and significantly more time investment for accurate counting. Due to the range of environments that these photos are taken in, the objects per photo vary significantly, roughly 80–500 objects per frame. With an average human count time being ∼60–180 s per photo, while MLDAAPP maintains a similar implementation time as described in *Object Counting Case #1*, with substantial potential time savings (Shahmohamadloo et al. 2025).

### Object tracking case #3: movement tracking and comparison with other CV approaches

In addition to photo analysis, MLDAAPP can help to employ YOLOv8 with a custom tracker to generate empirical data from videos (Figs. 1 and 3). We used 2 simplified standardized environments alongside 2 complex nonstandardized videos to examine the flexibility of MLDAAPP for video analysis. Additionally, we analyzed 2 videos from the web to further demonstrate the breadth over which MLDAAPP can be used (all videos available in Supplementary Table 1). MLDAAPP is effective in generating empirical data from videos with various challenges, including small and large object sizes, small and large object quantities, slow and fast object movement, low and high resolution, low and high frame rate, and simple or complex backgrounds (Supplementary Table 1).

We compared the performance of MLDAAPP to idTracker.ai, TRex, and DeepLabCut (Mathis et al. 2018; Moen et al. 2019; Romero-Ferrero et al. 2019; Lürig et al. 2021; Walter and Couzin 2021) across a range of input videos. Using shadowless, high frame rate videos of *Drosophila* from idTracker.ai, MLDAAPP, idTracker.ai, and TRex algorithms successfully tracked individuals with near 100% accuracy. DeepLabCut requires multiple reference points to learn about the object; therefore, small objects with little detail discernment are not feasible to use for training. We next compared performance on *Drosophila* videos collected with a lower cost DSLR setup (∼$700 in 2023) which produced videos with inconsistent shading and some visual noise. MLDAAPP tracked all individuals but idTracker.ai, TRex, were not capable of consistently tracking over half of the 17 *Drosophila* and both produced false positives (Fig. 3)—DeepLabCut was again unable to be trained. Finally, we used 2 videos (Supplementary Table 1 “Hamsters” & “Honey Bees”) generated by other parties that contained visual noise from shadows, inconsistent lighting, and object occlusion. Using the “Hamsters” input tracking performance of DeepLabCut was similarly successful to that of MLDAAPP, but was poor for both idTracker.ai and TRex with more false positives than individuals accurately identified and tracked (Fig. 3). idTracker.ai, TRex, and DeepLabCut require a static number of objects in input videos and hence were unable to run the “honey bees” video due to dynamic movement of objects in and out of frame.

## Discussion

The streamlined collection of unbiased phenotypic data is a limiting factor in many disciplines of biology, including genetics (McGill et al. 2006; Houle et al. 2010; Clark et al. 2020). The combination of ML and artificial intelligence holds considerable promise for the study of a wide range of organismal traits and the speed of innovation in this area is considerable (Krenn et al. 2022). Existing methods that use ML work well for inputs that are largely standardized ranging from cells to individuals (Mathis et al. 2018; Moen et al. 2019; Lürig et al. 2021). By applying exceptional computer vision and ML tools, including YOLOv8, the MLDAAPP package is both accurate and fast at translating photos and videos to phenotypic data for groups of individuals. The key to the increased flexibility and accuracy of MLDAAPP relative to existing programs is the YOLO algorithm's ability to learn general object representations (Redmon et al. 2016). This efficacy is largely maintained even with imperfect image and video inputs, making MLDAAPP uniquely affordable and flexible to implement. To reduce barriers to immediate usage, we provide protocols and pretrained algorithms for measures of *Drosophila* fecundity and movement. While we will continue to add functionality that enhances utility, MLDAAPP is a uniquely capable ML program for translating visual inputs to a wide range of data that are central to the study of populations. With open-source code and a growing number of users, MLDAAPP can contribute to solving “phenomics challenges” that are increasingly rate limiting in the study of ecology, evolution, and genetics (Houle et al. 2010).

## Supplementary Material

jkaf173_Supplementary_Data

## Data Availability

The data used in the protocol of this study are available through figshare (https://doi.org/10.25387/g3.29487929). Supplemental material available at G3 online.
